# A diagnostic model for predicting type 2 nasal polyps using biomarkers in nasal secretion

**DOI:** 10.3389/fimmu.2022.1054201

**Published:** 2022-12-21

**Authors:** Zaichuan Wang, Qiqi Wang, Su Duan, Yuling Zhang, Limin Zhao, Shujian Zhang, Liusiqi Hao, Yan Li, Xiangdong Wang, Chenshuo Wang, Nan Zhang, Claus Bachert, Luo Zhang, Feng Lan

**Affiliations:** ^1^ Beijing Key Laboratory of Nasal Disease, Beijing Institute of Otolaryngology, Beijing, China; ^2^ Department of Otolaryngology Head and Neck Surgery, Beijing TongRen Hospital, Capital Medical University, Beijing, China; ^3^ Department of Allergy, Beijing TongRen Hospital, Capital Medical University, Beijing, China; ^4^ Upper Airways Research Laboratory, Department of Otorhinolaryngology, Ghent University, Ghent, Belgium

**Keywords:** chronic rhinosinusitis with nasal polyps, diagnostic model, nasal secretion, IL-5, blood eosinophils

## Abstract

**Background:**

Predicting type 2 chronic rhinosinusitis with nasal polyps (CRSwNP) may help for selection of appropriate surgical procedures or pharmacotherapies in advance. However, an accurate non-invasive method for diagnosis of type 2 CRSwNP is presently unavailable.

**Methods:**

To optimize the technique for collecting nasal secretion (NasSec), 89 CRSwNP patients were tested using nasal packs made with four types of materials. Further, Th2^low^ and Th2^high^CRSwNP defined by clustering analysis in another 142 CRSwNP patients using tissue biomarkers, in the meanwhile, inflammatory biomarkers were detected in NasSec of the same patients collected by the selected nasal pack. A diagnostic model was established by machine learning algorithms to predict Th2^high^CRSwNP using NasSecs biomarkers.

**Results:**

Considering the area under receiver operating characteristic curve (AUC) for IL-5 in NasSec, nasal pack in polyvinyl alcohol (PVA) was superior to other materials for NasSec collection. When Th2^low^ and Th2^high^CRSwNP clusters were defined, logistic regression and decision tree model for prediction of Th2^high^CRSwNP demonstrated high AUCs values of 0.92 and 0.90 respectively using biomarkers of NasSecs. Consequently, the pre-pruned decision tree model; based on the levels of IL-5 in NasSec (≤ 15.04 pg/mL), blood eosinophil count (≤ 0.475*10^9^/L) and absence of comorbid asthma; was chosen to define Th2^low^CRSwNP from Th2^high^CRSwNP for routine clinical use.

**Conclusions:**

Taken together, a decision tree model based on a combination of NasSec biomarkers and clinical features can accurately define type 2 CRSwNP patients and therefore may be of benefit to patients in receiving appropriate therapies in daily clinical practice.

## Introduction

Chronic rhinosinusitis with nasal polyps (CRSwNP) is an inflammatory disease of the nasal and paranasal cavities, which can be distinguished from chronic rhinosinusitis without nasal polyps (CRSsNP), the other phenotype of CRS, according to patients’ symptoms and by endoscopy and computed tomography (CT) scan. However, following the “one-size-fits-all” treatment strategy in clinic, some CRSwNP patients with similar symptoms may have different therapeutic outcomes, suggesting that there may be different underling pathomechanisms (1). In this regard, endotyping helps in the recognition of the complexity and heterogeneity of disease and selection of appropriate surgical procedures and pharmacotherapy for patients (2).

Type 2 CRSwNP patients are characterized by the presence of staphylococcal enterotoxin immunoglobulin E (SE-IgE) and type 2 cytokines such as IL-4, IL-5 and IL-13, as well as high asthma comorbidity and recurrence (3–5). Indeed in general, non-type 2 CRSwNP and type 2 CRSwNP require different pharmacological, surgical approaches and biologic treatments (6). “Reboot” approach or extensive surgery, which is not classical functional endoscopic sinus surgery, can significantly reduce the recurrence of type 2 CRSwNP by removing the dysfunctional and inflamed mucosa without interfering with re-epithelialization of sinus wall (2, 7). Biologics targeting antigen-specific IgE, IL-5 or IL-4 receptor are able to control symptoms and complications of severe type 2 CRSwNP (1, 8–10). Thus, distinguishing type 2 CRSwNP from non-type 2 CRSwNP is likely to benefit specialists in selecting appropriate effective treatments to improve the outcome in patients.

Whilst tissue inflammatory molecular and cellular biomarkers are generally used to distinguish type 2 CRSwNP from non-type 2 CRSwNP patients, a precise technique for diagnosing type 2 CRSwNP prior to treatment is still not available (4, 11–13). In this regard the European Forum for Research and Education in Allergy and Airway Diseases (EUFOREA) (14) has described an algorithm based on comorbid asthma and blood eosinophil count, which can define type 2 CRSwNP in Europe with 86.4% sensitivity and 72.7% specificity. However, inflammatory signatures of CRSwNP vary around the world, and a lower ratio of comorbid asthma is noticed in China, Korea and Thailand compared to Europe (15). Thus, whether the EUFOREA (14) recommended algorithm can be used for type 2 CRSwNP patients in Asian countries awaits further investigations. Assessment of local inflammation in nasal secretion (NasSec) or nasal mucus may be a useful method in the diagnosis of type 2 CRSwNP. Reportedly, assessment of local inflammation in NasSec obtained from common meatus or nasal mucus was shown to be non-invasive and reproducible for local immunological analysis (16). Furthermore, inflammatory markers in NasSecs have been used to evaluate the efficacy of biologics in CRSwNP patients receiving Dupilumab treatment (17). However, current sampling techniques to obtain NasSec or nasal mucus vary widely with regard to the material and preparation of the sample for assessment of inflammatory markers; with reference ranges for inflammatory mediators also not been elucidated for routine clinical use (18–21).

In this study, we therefore attempted to standardize the process for NasSec collection from CRSwNP patients, with particular reference to the optimal method for collection. Furthermore, we attempted to establish a diagnostic model for routine clinical use, based on the patient’s clinical features and biomarkers of local inflammation in NasSec, to accurately define type 2 CRSwNP patients prior to surgery.

## Materials and methods

### Patients and study design

Patients with CRSwNP scheduled to undergo nasal surgery were recruited into the study from the Department of Rhinology and Allergy, Beijing TongRen Hospital from June 2019 to May 2021. The diagnosis of CRSwNP was made according to the European Position Paper on Rhinosinusitis and Nasal polyps 2012 guidelines (22). Patients with cystic fibrosis, fungal sinusitis, or primary ciliary dyskinesia were excluded. The diagnosis of asthma was confirmed by pulmonologists according to symptoms and respiratory function tests. Patients were considered to be allergic based on the presence of serum specific IgE to inhalant allergens, including Dermatophagoides pteronyssinus (*Der p*), Dermatophagoides farinae (*Der f*), Mugwort, Penicillium notatum, Candida albicans, Alternaria, Cladosporium, and Aspergillus. None of the patients had taken oral or intranasal glucocorticoids or antibiotics within 4 weeks before surgery. Twice-daily nasal irrigation and mucolytics were used for patients to alleviate clinical symptoms if necessary, and leukotriene modifiers and antihistamines were also prescribed to CRSwNP patients with allergy. Prior to surgery, NasSec and blood samples were collected from each CRSwNP patient without acute infection, and nasal polyp tissues were collected during surgery; and all samples were stored by freezing at -80°C until further analysis. This study was approved by the Ethics Committee of Beijing TongRen Hospital (TRECKY2019-027), and written informed consent was obtained from all patients before enrolment in the study.

Subjects were assigned to two cross-sectional groups. The clinical characteristics are presented in **Table 1**. In brief, 89 patients were included in Group 1 and were randomized to receive one of the four nasal packs for NasSec collection to evaluate the appropriate nasal pack for NasSec collection. Based on that, nasal secretion of 142 patients in Group 2 was collected using the appropriate material (PVA, as mentioned in results section), in the meanwhile, histologic samples were obtained for further analysis. To estimate appropriate nasal pack for NasSec collection, the sample size was calculated using linear correlation analysis (correlation coefficient = 0.7), and in developing a diagnostic model (machine learning), area under the curve (AUC) was regarded as the main index when calculating the sample size.

**Table 1:** Patient characterization.

	Group 1 (N=89)	Group 2 (N=142)
Sex, (male/female)	65/24	103/39
Age, (years), median (range)	50.0 (38.0, 58.0)	47.0 (35.0, 55.0)
Asthma, No. (%)	20 (22.5%)	47 (33.1%)
Revision surgery, No. (%)	25 (28.1%)	44 (31.0%)
Aspirin-exacerbated respiratory disease, No. (%)	3 (3.4%)	7 (4.9%)
Biomarker in nasal polyp tissue		
IL-5 (pg/mL), median (range)	61.9 (12.2, 214)	195 (104, 351)
Total IgE (KU/L), median (range)	207 (101, 350)	107 (15.2, 359)

### Clinical features of CRSwNP patients

The clinical characteristics documented for these patients included nasal symptoms, body mass index (BMI), endoscopic and CT assessment, peripheral blood cell counts, histological evaluation and grading eosinophilia in nasal smears as follows: i. The nasal symptoms were determined using visual analogue scale (VAS) score. Each symptom was marked by patients on a 10-cm scale. Zero means no discomfort and 10 means the most unbearable symptoms. ii. BMI was calculated as the weight in kilograms divided by the square of the height in meters for each patient. iii. Bilateral nasal polyp score was evaluated according to the 5-point scale (23). The overall CT score was determined according to the Lund-Mackay staging system (24); and the ratio of total ethmoid sinus score to total maxillary score (E/M ratio) was also calculated. iv. A complete blood cell count in CRSwNP patients without infection was performed before surgery; and nasal smears collected from the middle portion of the inferior turbinate in both nostrils were stained with May-Grünwald-Giemsa stain for assessment of eosinophils by light microscopy. v. Polyp tissue sections were stained with hematoxylineosin (H&E), eosinophils, neutrophils, plasma cells and lymphocytes were assessed by bright-field light microscopy (Olympus, BX51) at ×400 magnification. Immune cells were counted in 3 non-overlapping fields in the lamina propria by two pathologists. The numbers of inflammatory cells were expressed as the percentage of total inflammatory cells present. vi. Eosinophilia in nasal smears were graded according to the literature (25), resulting in scores of 0 to 4: 0, none eosinophils; 0.5, occasional eosinophils, 1, 1-5 eosinophils/high power field (HPF) or small eosinophils clumps; 2, 5-15 eosinophils/HPF or larger eosinophils clumps; 3, 5-15 eosinophils/HPF or clumps of eosinophils that do not cover the entire field; 4, >20 eosinophils/HPF with clumps covering the entire field.

### NasSec collection

NasSec were collected using four types of commercially available nasal packs of the same size (2.5 cm *0.5 cm *0.67 cm) and made from different materials; including polyvinyl acetate (PVAc, IVALON, Fabco^®^, New London, CT, USA), cross-linked polyvinyl alcohol (cl-PVA, MEROCEL^®^, Medtronic, Florida, USA), polyvinyl alcohol (PVA, JCMED, Tianjin, China), and polyvinyl fluoride (PVF, Kuaijie, Beijing YINGJIA Co., Beijing, China). Generally, all nasal packs were made from organic polymers. PVA is a water-soluble synthetic polymer; PVAc is the precursor for PVA with poor water absorption; PVF is made from PVA by reaction with formaldehyde with the feature of water resistance and solubility of non-polar solvents; cl-PVA is a PVA sponge with polyethylene film. Prior to collection of NasSec, the nasal cavity was cleaned by wiping with cotton wool soaked in 0.9% sodium chloride (NaCl) to clear any pre-secreted secretions, and then the specified nasal pack was visually placed between the inferior turbinate and the septum in each nostril for 5 min without local or general anaesthesia. No adverse effects or intolerance were reported. Afterwards, the nasal pack was carefully retrieved using forceps and transferred into 15 mL tube containing 3 mL 0.9% NaCl, for 2 hours at 4°C to dissolve the NasSec in the saline as indicated by Berings and colleagues (19). At the end of this period, the contents of the tube were transferred into a 5 mL syringe and the secretion was recovered from the nasal pack using the plunger, followed by centrifugation at 1500 g at 4°C for 15 min. The supernatant from each sample was collected and aliquoted before storage at -80°C until further analysis. To evaluate the standard IgE recovery rate of different nasal packs, different doses (10, 20, 40, 80, 160, 320, 640 ng/mL) of standard IgE (human IGHE, Sino Biological, Beijing, China) were diluted into 3mL 0.9% NaCl. And then nasal packs were merged into standard IgE solution for 2h, IgE was measured in the solution after centrifuge.

### Measurement of inflammatory markers

Inflammatory cytokines were measured in nasal tissue and NasSec samples. Prior to assessment, nasal polyp tissues were suspended in 0.9% NaCl solution with a complete protease inhibitor cocktail (Roche, Mannheim, Germany) based on tissue weight, and homogenized mechanically using a Tissue Lyser LT (Qiagen, Hilden, Germany), as described previously (26). The presence of total IgE and eosinophil cationic protein (ECP) was measured in tissue homogenates, NasSecs or standard IgE in 0.9%NaCl using the ImmunoCAP system (Immunodiagnostics; Thermo Fisher Scientific, Uppsala, Sweden), according to manufacturer’s instructions. The inflammatory markers IL-5, CCL5, CCL24, CCL26, IFN-γ, IL-17 and periostin were assessed by Luminex xMAP suspension array technology (R&D, Minneapolis, Minnesota, USA) in a Bio-Plex 200 system (Bio-Rad, MI). The cut off value of cytokines was defined according to half of the detection limit of commercial kit as described in the literature (11) (0.27 pg/mL for IL-5). Concentrations of the biomarkers in tissue homogenates were expressed as mass versus volume after multiplication by a homogenization dilution factor of 11. Values for total IgE ≥ 112.2 KU/L in tissue were regarded as positive (4).

### CRSwNP patients clustering by Partitioning Around Medoids algorithm

Tissue inflammatory biomarkers such as IL-5, ECP and total IgE in polyp tissue, comorbid-asthma, peripheral blood eosinophil count, and revision surgery were used for type 2 CRSwNP clustering analysis as documented in the literature (27–29). Continuous variables were transformed to Z-score, while categorical variables were expressed as 0 or 1, and a ‘gower’ dissimilarity matrix, which allows both continuous and categorical data, was calculated. The subjects were sorted into groups using the PAM algorithm, with cluster numbers ranging from 2 to 15 generated. The optical number of clusters was determined by the “elbow” (maximum change) in the wss plot or by the maximized average silhouette (**Supplementary Figure 1**). To visualize distinction among clusters, principal component analysis (PCA) was performed by plotting subjects in first two dimensions after multidimensional scaling. A modified heat-map was used for descriptive characterization of individual clusters.

### Machine learning

To establish a prediction model for type 2 CRSwNP model, two common machine learning algorithms (decision tree and logistic regression) were used as described before (30). Eighteen clinical features and biomarkers, as described in **Table 2**, were used for machine learning. Missing values were imputed using the mean value from 5 nearest neighbouring values (31). The naive random over sampling method was applied to adjust for imbalance in two groups. For logistic regression model, the least absolute shrinkage and selection operator (LASSO) regulation was performed for feature selection. 5-folds cross-validation was implemented to avoid overfitting. We used the AUC to evaluate the performance of the model and the model in which the cross-validation AUC was within one standard error of the maximum was selected. When λ = 0.052, 5 factors were screened out by the logistic regression model; including IL-5 and CCL26 in nasal secretion, comorbid asthma, revision surgery and blood eosinophil count; exhibiting a high goodness-of-fit in the validation dataset (cross-validation AUC = 0.90, **Supplementary Figures 2A, B**). Each decision node of a decision tree corresponds to a single predictor variable and a split cut-off on that variable. The cut-off of variables was determined according to the minimized Gini index when building the decision tree. Gini index formula was $gini\;index=1-P_{1}^{2}-P_{2}^{2}$ , which *P*_1_ and *P*_2_ represent the proportion of Th2^high^ CRSwNP in two subcategories. Pre-pruning was used to modify the decision tree model by limiting the leaf nodes. Optimal leaf nodes were generated based on the AUC value in 5-folds cross-validation. Model with 4 leaf nodes was selected, showing a high cross-validation AUC (0.89, **Supplementary Figure 2C**). Machine learning was performed using the Scikit-learn, imbalanced-learn and glmnet package.

**Table 2:** Demographic and clinical characteristics of CRSwNP patients investigated for development of the predicting model.

Group 2	Th2^low^	Th2^high^	*p*-value
	(N=55)	(N=87)	
Sex (male/female), †	44/11	59/28	0.28
Age (years), median (range) †	48.0 (35.0, 56.5)	46.0 (35.0, 55.0)	0.86
BMI, median (range) †	25.4 (22.2, 27.5)	24.7 (22.7, 26.7)	0.427
Present smoking, No. (%) †	11 (20.0%)	22 (25.3%)	0.624
Asthma, No. (%) †	0 (0%)	47 (54.0%)	<0.001
Revision surgery, No. (%) †	21 (38.2%)	23 (26.4%)	0.335
VAS score			
Nasal blockage, median (range)	5.4 (4.3, 6.4)	5.9 (4.5, 6.7)	0.055
Nasal discharge, median (range)	4.6 (3.2, 5.7)	4.6 (3.8, 5.8)	0.714
Headache and facial pain, median (range)	1.6 (0, 5.2)	0.8 (0, 3.6)	0.117
Smell loss, median (range)	4.8 (3.5, 6.5)	5.90 (4.9, 9.1)	0.002
Blood sampling			
White blood cells (10^9^/L), median (range) †	6.59 (5.12, 7.44)	6.67 (5.33, 7.60)	0.633
Absolute count of eosinophils (10^9^/L), median (range) †	0.200 (0.130, 0.335)	0.380 (0.275, 0.550)	<0.001
Proportion of eosinophils (%), median (range)	3.30 (1.95, 5.25)	5.70 (4.35, 8.25)	<0.001
IgE (KU/L), median (range)	68.0 (27.2, 164)	143 (62.0, 305)	0.057
Bilateral nasal polyp score, median (range)	4(3-5)	4(3-5)	0.726
CT assessment			
Lund-Mackay total score, median (range) †	15.0 (12.0, 21.0)	18.0 (13.0, 21.0)	0.262
E/M ratio, median (range) †	2.00 (1.67, 2.67)	2.00 (2.00, 3.50)	0.17
Missing	2 (3.6%)	2 (2.3%)	
Biomarker in nasal polyp tissue			
IL-5 (pg/mL), median (range)	14.4 (2.97, 99.9)	212 (62.3, 548)	<0.001
ECP (pg/mL), median (range)	759 (203, 1610)	3060 (2490, 3530)	<0.001
IgE (KU/L), median (range)	97.9 (67.7, 189)	261 (162, 463)	<0.001
IFN-γ (pg/mL), median (range)	10.1 (10.1, 27.2)	10.1 (10.1, 27.2)	0.555
IL-17 (pg/mL), median (range)	5.50 (5.50, 33.4)	5.50 (5.50, 8.64)	0.046
Histological evaluation			
Eosinophils (%), median (range)	29.3 (7.7, 56.1)	54.2 (40.7, 73.7)	<0.001
Neutrophils (%), median (range)	2.1(0.8, 8.3)	1.4 (0.6, 6.1)	0.458
Plasma cells (%), median (range)	10.7 (7.5, 23.4)	9.1 (5.1, 17.6)	0.057
Lymphocytes (%), median (range)	47.0 (21.4, 59.6)	22.8 (14.7, 36.6)	<0.001
Biomarker in nasal secretion			
IL-5 (pg/mL), median (range) †	0.890 (0.270, 4.37)	6.59 (1.30, 30.2)	<0.001
ECP (pg/mL), median (range) †	30.0 (10.9, 80.4)	56.5 (19.1, 131)	0.011
IgE (KU/L), median (range) †	5.69 (4.80, 9.90)	18.6 (7.89, 28.9)	<0.001
CCL5 (pg/mL), median (range) †	66.2 (38.2, 201)	61.0 (16.7, 130)	0.359
CCL24 (pg/mL), median (range) †	323 (149, 590)	409 (220, 701)	0.259
CCL26 (pg/mL), median (range) †	15.0 (7.93, 56.0)	53.1 (14.0, 139)	0.0124
Periostin (pg/mL), median (range) †	20572 (7411, 35765)	33759 (15391, 46870)	0.0719
IFN-γ No. positive (positive ratio)	13 (23.6%)	26 (29.9%)	0.584
IL-17 No. positive (positive ratio)	17 (30.9%)	23 (26.4%)	0.704
Grade of eosinophilia in nasal smears, median (range)†	0 (0, 1)	1 (0, 2)	0.098
Missing cases of nasal smears	1 (1.8%)	8 (9.2%)	

Continuous variables are expressed as medians and interquartile ranges, while categorical variables are expressed as frequency and percentage. BMI, body mass index; CT, computed tomography; E/M ratio, ratio of Lund-Mackay scores for the ethmoid sinus and maxillary sinus; HPF, high power field; VAS, visual analogue scale; †, features used in machine learning.

### Statistical analysis

The Chi-square or Fisher’s exact tests were used for comparing categorical variables. Continuous variables were analyzed by the Mann-Whitney U test or Kruskal-Wallis H test. A Benjamini-Hochberg tail-area-based correction for multiple comparisons was applied to strictly control for false positive. Correlations for cytokine concentrations between NasSec and tissue homogenate were analyzed by Pearson correlation and p value less than 0.05 was considered statistically significant. The diagnostic ability of each biomarker or predicting model was calculated based on the AUC. Statistical analysis was performed using Python programming language (version 3.8.8, Python Software Foundation) and R software (version 4.0.3, R Development Core Team). Data were plotted using the ggplot2 and pROC package for R.

## Results

### NasSec collected by PVA nasal pack can be used for immune assessments in CRSwNP patients

**Figure 1:**
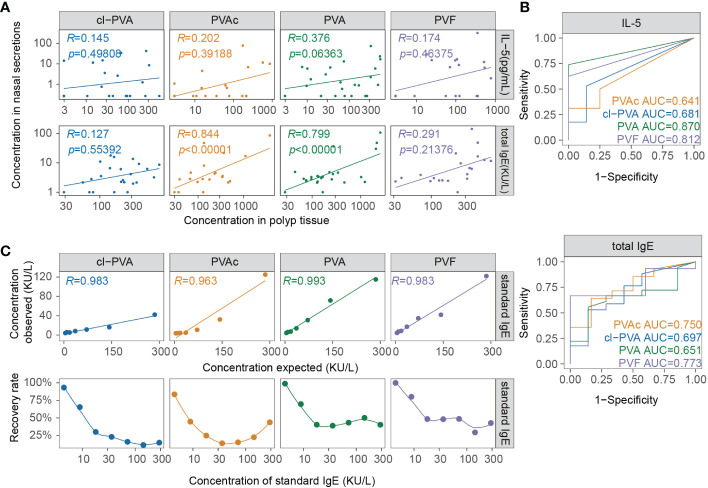
Comparison of IL-5 and IgE levels in nasal secretions collected by 4 types of nasal packs. **(A)** Correlation between total IgE and IL-5 (Group1, n = 89) in nasal secretion and tissue collected by 4 types of nasal packs. **(B)** Receiver operating characteristic curves of IL-5, total IgE in nasal secretion (Group1, n = 89), using concentrations in tissue homogenate as gold standards. **(C)** Correlation and recovery rate of standard IgE in nasal secretion collected by four types of nasal packs. PVA, polyvinyl alcohol; PVAc, polyvinyl acetate; cl-PVA, cross-linked PVA; PVF, polyvinyl fluoride.

NasSec was collected from 89 CRSwNP patients (Group 1) using four different nasal packs. The clinical characteristics of patients were shown in **Table 1**. Association assessments indicated that total IgE in tissue homogenate is positively correlated to that in NasSec collected by PVA and PVAc (**Figure 1A**). Nevertheless, there was a close relationship between IL-5 in tissue homogenate and IL-5 in NasSec only collected by PVA (r=0.376; *p*=0.0636) (**Figure 1A**). In terms of total IgE measurement, the AUCs of NasSec collected by PVF (AUC=0.773) were superior to PVA (AUC=0.651) (**Figure 1B**). When comparing the sensitivity and specificity of IL-5 measurement in NasSec, PVA was found to be superior to other materials for NasSec collection (AUC=0.870) (**Figure 1B**). Due to the low sensitivity and specificity of total IgE measurement in NasSec, we also took different doses of standard IgE in 0.9% NaCl as positive controls to clarify the recovery rate of IgE using four nasal packs. As shown in **Figure 1C**, both PVA and PVF had higher recovery rates of standard IgE than PVAc and cl-PVA. Taking together, PVA was selected for the appropriate nasal packs for NasSec collection to evaluate type 2 inflammation.

### Type 2 CRSwNP patients defined by clustering analysis using tissue inflammatory biomarkers

**Figure 2:**
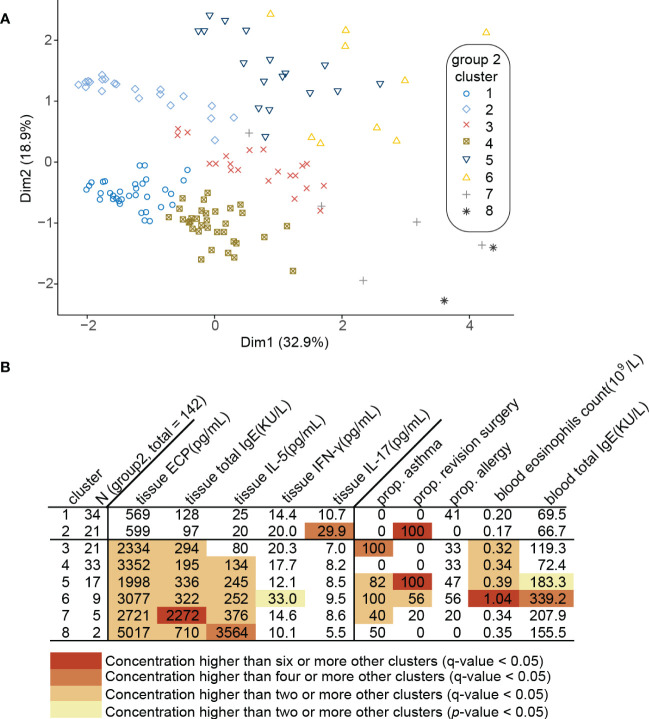
Clustering of 142 CRSwNP patients (Group 2). **(A)** Principal component analysis **(**PCA) plot of patients plotted in a 2-dimensional space after multidimensional scaling. **(B)** Modified heat map of data relevant to each cluster. Rows define clusters of patients with CRSwNP. Categorical variables (asthma, allergy and revision surgery) are shown as positive ratio. Continuous variables (tissue biomarkers, blood eosinophils count and blood total IgE) are shown as geometric mean. Each q-value represents Benjamini-Hochberg adjusted *p*-value.

The clinical characteristics of patients in Group 2 are shown in **Table 1**. Clustering 142 CRSwNP patients based on their tissue inflammatory biomarkers resulted in 8 separate clusters (**Figure 2A**). Clusters 1 and 2 were characterized by a low or undetectable concentration of IL-5, ECP and total IgE, and were thus designated as Th2^low^CRSwNP (**Figure 2B**). In clusters 3 to 8, CRSwNP patients with highest levels of type 2 biomarkers were regarded as Th2^high^CRSwNP (**Figure 2B**). Among the 8 clusters, CRSwNP patients in cluster 2 expressed an elevated level of tissue IL-17 and a high proportion of patients had revision surgery. Cluster 6 demonstrated the highest level of local type 2 biomarkers and type 2 systemic features such as high blood eosinophils count, serum IgE, and proportion of comorbid asthma and allergy (**Figure 2B**). Generally, the scores of nasal blockage and smell loss were higher in Th2^high^CRSwNP patients than that in Th2^low^CRSwNP patients. As expected, polyp tissue of Th2^high^CRSwNP patients had more eosinophils and fewer lymphocytes in comparison to the tissue of Th2^low^CRSwNP patients (*p*<0.001, **Table 2**).

### NasSec IL-5, NasSec total IgE, comorbid asthma and blood eosinophils counts are useful markers for diagnosis of Th2^high^CRSwNP

**Figure 3:**
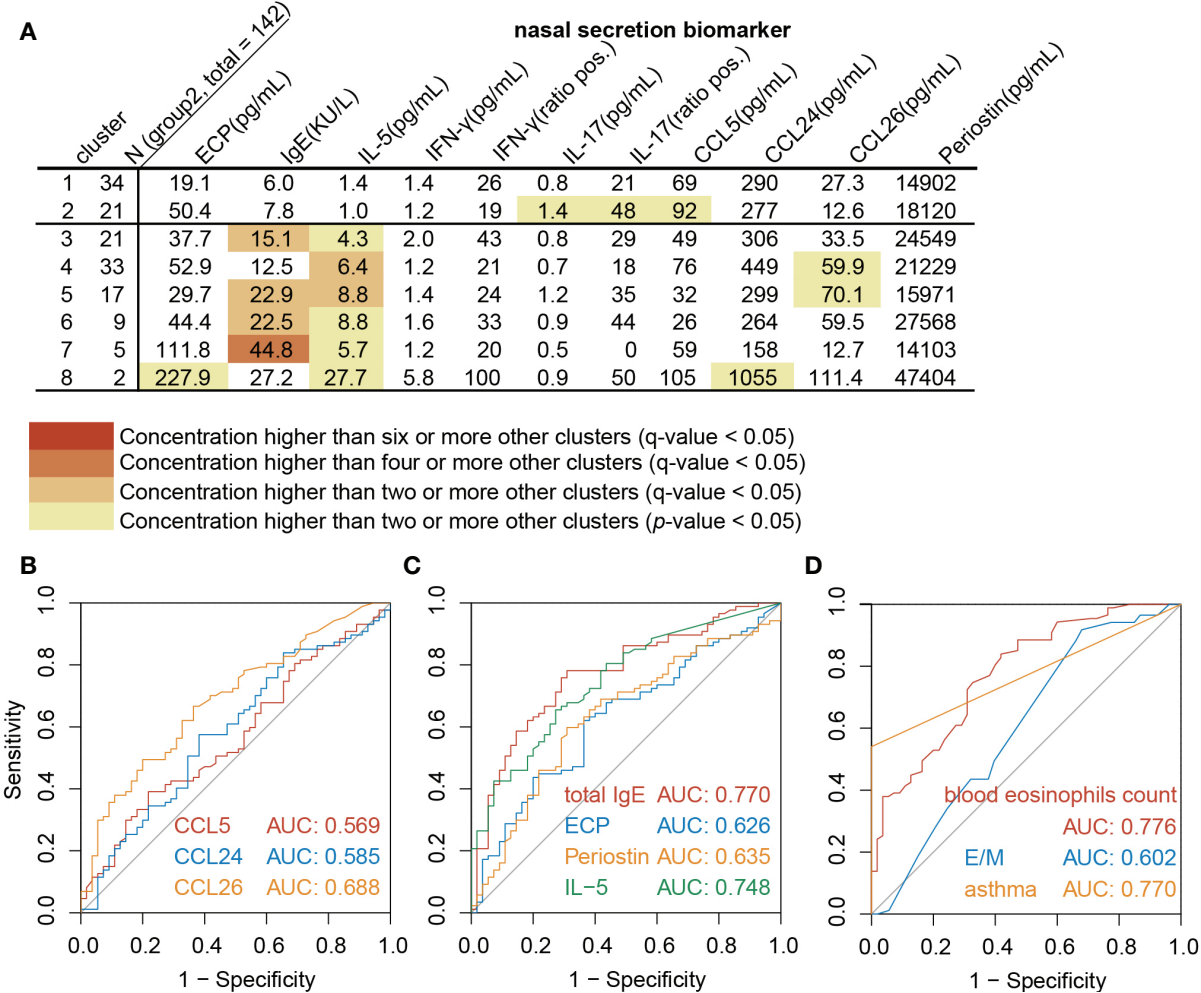
NasSec and clinic markers for diagnosis of Th2^high^CRSwNP. **(A)**. Modified heat map of biomarkers in nasal secretions from each cluster (Group 2, n = 142). **(B-D)**. Receiver operating characteristic curve (ROC) analysis for prediction of Th2^high^CRSwNP (Group 2, n = 142). AUC: area under ROC curve; E/M: ratio of Lund-Mackay scores for the ethmoid sinus and maxillary sinus.

Th2^high^CRSwNP patients in Beijing were predicted following the same algorithm recommended by EUFOREA (14), which confirmed the sensitivity and specificity to be 85.1% and 69.1% respectively (**Supplementary Figure 3**). Considering that, biomarkers in NasSec were included in to improve the accuracy of type 2 CRSwNP patient definition. Assessment of the inflammatory biomarkers analyzed in NasSec in different CRSwNP clusters (Group 2) further demonstrated the concentrations of IL-5 to be consistently significantly elevated in all Th2^high^ clusters (cluster 3 to 8), but not in Th2^low^ clusters (cluster 1 to 2) (**Figure 3A**). ROC analysis further demonstrated that NasSec IL-5, NasSec total IgE, comorbid asthma and blood eosinophils counts were good predictors of Th2^high^CRSwNP (**Figures 3B-D**).

### A decision tree prediction model using NasSec IL-5 in combination with comorbid-asthma and blood eosinophil count can accurately predict Th2^high^CRSwNP

**Figure 4:**
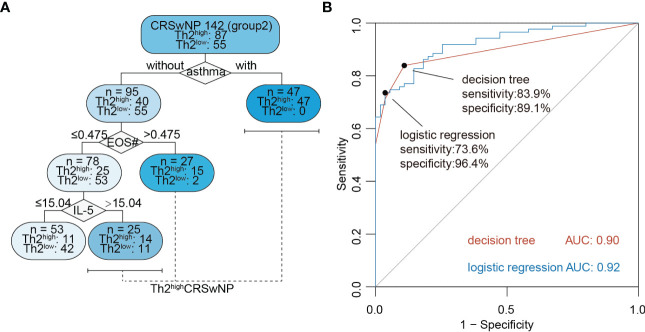
Machine learning models for non-invasive diagnosis of Th2^high^CRSwNP. **(A)** The decision tree model for predicting type 2 CRSwNP (Group 2, n = 142); IL-5 = concentration of the cytokine in nasal secretion collected by polyvinyl alcohol (PVA) nasal packs (pg/mL). EOS# = peripheral blood eosinophil absolute count (10^9^/L). **(B)** Receiver operating characteristic curve (ROC) analysis of logistic regression and decision tree model for prediction of Th2^high^CRSwNP (Group 2, n = 142).

In order to better predict Th2^high^CRSwNP using biomarkers in NasSec, two machine learning models were established based on clinical features and biomarkers in Group 2. A logistic regression model was expressed as y = 0.0070 × IL-5 in NasSec (pg/mL) + 0.00138 × CCL26 in NasSec (pg/mL) + 2.73 × asthma + 2.44 × blood eosinophil count (10^9^/L) – 0.3 × previous sinus surgery (with presence of asthma as 1 and absence of asthma as 0; with previous sinus surgery as 1 and without previous sinus surgery as 0); and a decision tree model was established as shown in **Figure 4A**. Considering the relatively low sensitivity of the logistic regression model (73.6%), the pre-pruned decision tree was finally selected as the preferred prediction model; with an absence of asthma comorbidity plus blood eosinophil count ≤ 0.475*10^9^/L and IL-5 concentration ≤ 15.04 pg/mL in NasSec shown to be suitable for distinguishing Th2^low^CRSwNP patients from Th2^high^CRSwNP patients. Furthermore, the AUC value for this prediction model was 0.90, with sensitivity and specificity of 83.9% and 89.1%, respectively (**Figure 4B**).

## Discussion

This study has demonstrated that Th2^high^CRSwNP patients can be identified by cluster analysis using reported clinical variables and biomarker cytokines. Using these parameters, we demonstrated for the first time that a decision tree based on presence or absence of comorbid asthma, the number of blood eosinophils and IL-5 concentration in NasSec collected by PVA nasal packs provides an accurate way of distinguishing Th2^high^CRSwNP patients from Th2^low^CRSwNP patients prior to treatment.

Indeed, NasSec is easier to obtain and a good non-invasive alternative to nasal tissue in assessing local inflammation. However, the biomarkers in nasal secretions and in nasal tissue are not always consistent (32). The techniques for collection and processing of NasSec vary widely and may influence the final analysis. In this regard, this study is the first to validate the common meatus NasSec collection procedure by comparing the suitability of four types of commonly employed nasal packs in Group 1 patients and to demonstrate that nasal packs using PVA were the most efficient for collecting NasSec for analysis of the inflammatory cytokine IL-5. In terms of total IgE, the NasSec collected by PVA and PVAc was high correlated with that in nasal tissue, while PVA and PVF had higher recovery rates of standard IgE. Thus, in light of this evidence, PVA was eventually chosen as the most appropriate material for collection of NasSecs to evaluate local inflammation.

The AUC of the algorithm recommended by EUFOREA (14) to predict type 2 CRSwNP in China was found to be 0.85, similar to that in Europe. However, the specificity (69.1%) of this algorithm is relatively low. Although we cannot parallelly compare our 8 clusters to already known clusters described in the literature (11) due to the exclusion of CRSsNP patients during the subject enrollments. Still, we noticed that a Th2^low^CRSwNP cluster (cluster 2) in our study has a high recurrence rate, which is similar to the described Th2^low^CRSwNP clusters in Tomassen et al.’s study (11). Among all Th2^high^CRSwNP clusters (cluster 3-8), patients in cluster 7 and 8 are characterized by a higher level of IL-5, ECP and total IgE, while 92% of patients in Th2^high^CRSwNP clusters are less type 2 inflammation. It is further evident that the inflammatory signatures of CRSwNP varied in the whole world. Thus, to establish an easy-to-use diagnostic model with higher specificity, we included selected a variety of inflammatory cytokines, CCL26, CCL5 and periostin in NasSec, CT score, and useful clinical features as variables for type 2 CRSwNP diagnosis in our model. Our data demonstrated that the use of NasSec IL-5 alone or peripheral blood eosinophil count could assess nasal type 2 inflammation (AUC for IL-5 alone = 0.748; AUC for blood eosinophil count = 0.776). Eventually, a pruned decision tree model, which provided a higher AUC (0.90), was therefore established to predict type 2 CRSwNP accurately.

This study is, however, somewhat limited in that the selection of the specific inflammatory cytokines for identification of type 2 CRSwNP was generally based on the data available in the literature, and that these cytokines were mainly evaluated in the nasal secretion for type 2 identification. In particular, some typical type 2 related biomarkers such as IL-4, IL-13 and alarmins were not included in the present study as IL-4 and IL-13 are not easily detectable in NasSec or tissue of same type 2 CRSwNP patients. Furthermore, as only CRSwNP patients from Beijing were enrolled in this study, the applicability of our findings to other regions in the world remains to be elucidated.

## Conclusion

This is the first study to compare the suitability of different materials in nasal packs for collection of NasSec, and to demonstrate that PVA appears to be the most appropriate material in this regard. This study has further demonstrated that with comorbid asthma, the number of blood eosinophils > 0.475*10^9^/L or IL-5 in NasSec > 15.04 ng/mL can be used to define Th2^high^CRSwNP. This may be of particular clinic relevance because the ability to accurately diagnose type 2 CRSwNP or non-type 2 CRSwNP prior to commencement of treatment may aid in the selection of appropriate surgical procedures and pharmacotherapy accordingly.

## Data availability statement

The raw data supporting the conclusions of this article will be made available by the authors, without undue reservation.

## Ethics statement

The studies involving human participants were reviewed and approved by the Ethics Committee of Beijing TongRen Hospital (TRECKY2019-027), and written informed consent was obtained from all patients before enrollment in the study.

## Author contributions

FL and LuZ conceived and designed the experiments. SD, CW and XW collected and handled the samples. QW, YZ, LiZ, and SZ performed the experiments. SD, QW, FL, LH and ZW analyzed the data. SD, FL, QW, ZW, NZ, CB, LiZ and LuZ drafted and revised the manuscript. All authors contributed to the article and approved the submitted version.
