# Supplementary material for: A diagnostic model for predicting type 2 nasal polyps using biomarkers in nasal secretion
Source: Front Immunol. 2022 Dec 21;13:1054201. doi: 10.3389/fimmu.2022.1054201 (PMC9811186; doi:10.3389/fimmu.2022.1054201)

Supplementary Material

## Supplementary Figures

**Supplementary Figure 1.** Estimating the optimal number of clusters (k). **A.** The variance within the clusters decreases as cluster increases, but an elbow can be seen at k = 8 (Group 2, n = 142). **B.** Average silhouette width maximized when k = 8 (Group 2, n = 142).


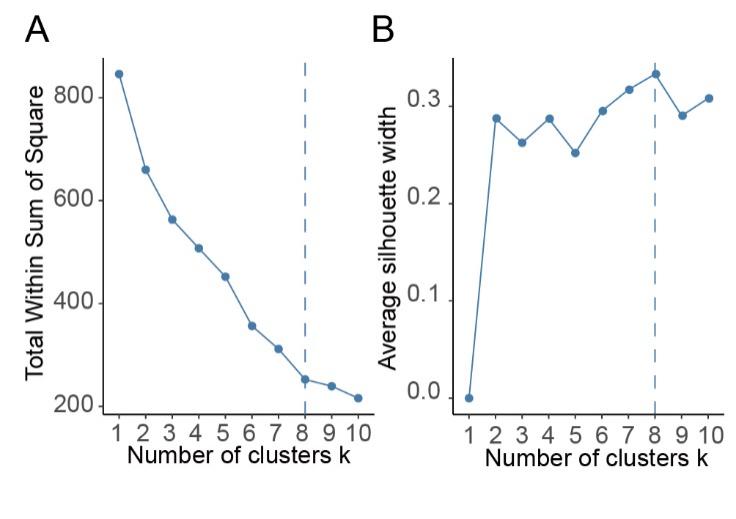


**Supplementary Figure 2:** Cross validation of machine learning models of Group 2 CRSwNP patients. **A.** Select the λ that gives the most regularized model for the least absolute shrinkage and selection operator (LASSO) logistic regression. λ = 0.052 was selected, when cross-validation AUC = 0.90. **B.** Coefficient of each variable in logistic regression model. When λ = 0.052, 5 variables were selected. **C.** Pre-pruning by limiting the split nodes for decision tree. 4 leaf nodes were selected, with the mean cross-validation AUC of 0.89. AUC: area under receiver operating characteristic curve.

**
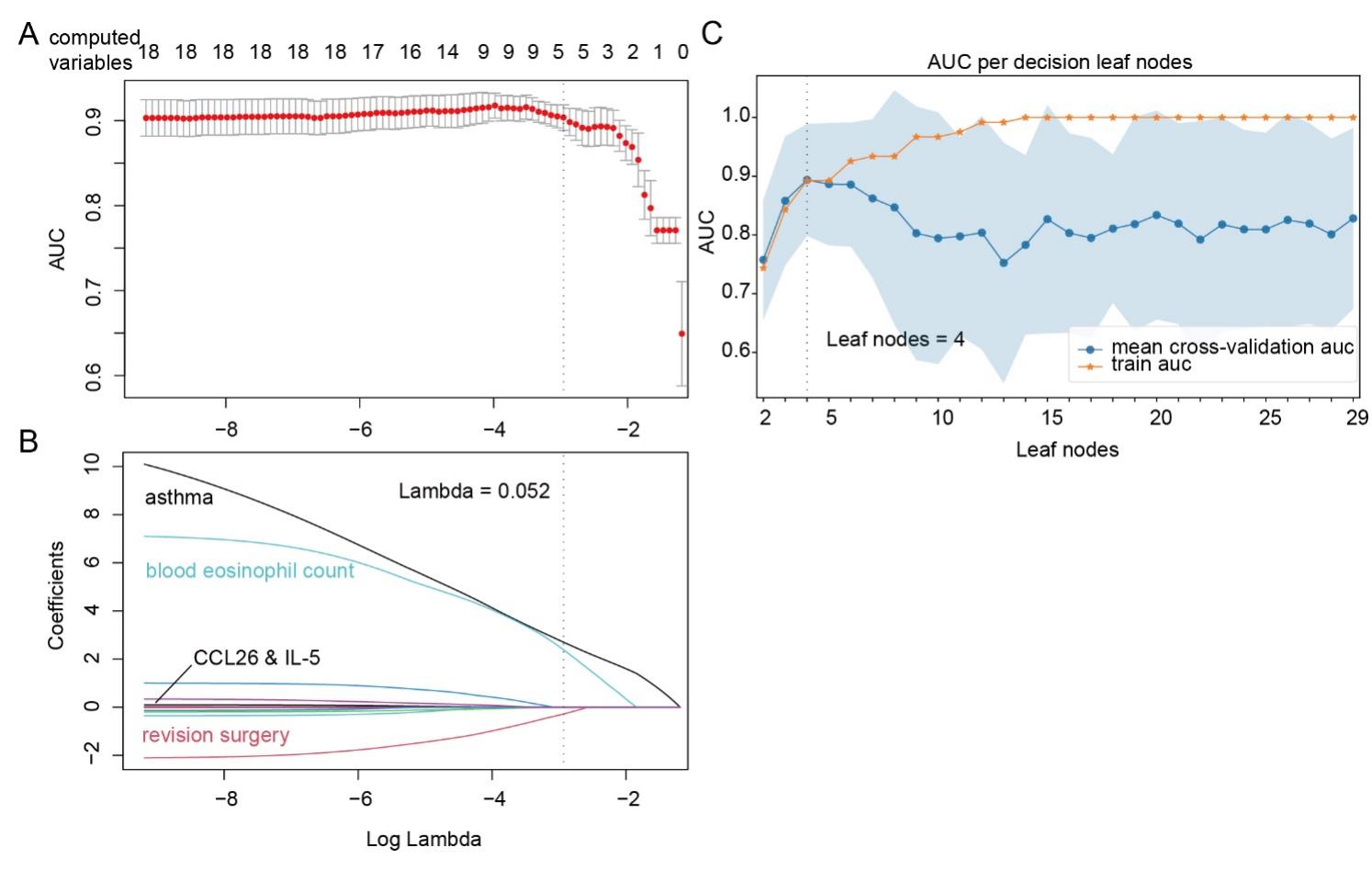
**

**Supplementary Figure 3:** Verify the accuracy of algorithm recommend by the European Forum for Research and Education in Allergy and Airway Diseases (EUFOREA) in Group 2 CRSwNP tissue from Beijing.


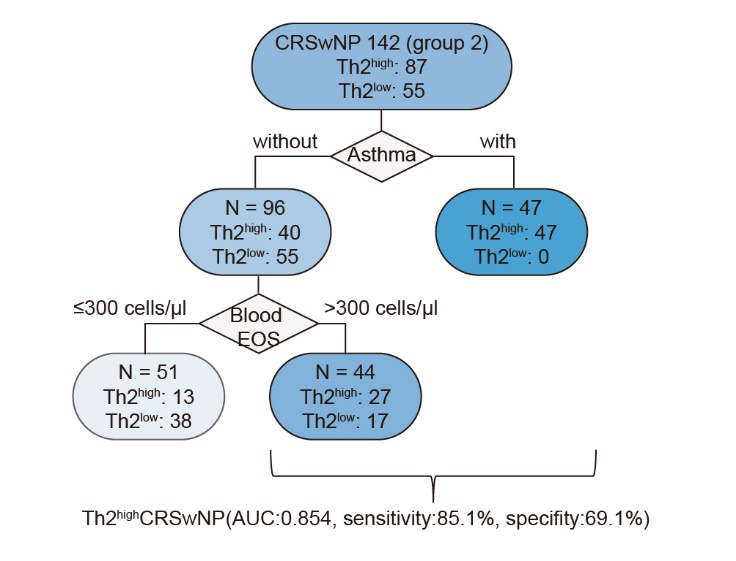

Supplement: Supplementary file 1 [file DataSheet_1.docx]
